# Differential association of air pollution exposure with neonatal and postneonatal mortality in England and Wales: A cohort study

**DOI:** 10.1371/journal.pmed.1003400

**Published:** 2020-10-20

**Authors:** Sarah J. Kotecha, W. John Watkins, John Lowe, Jonathan Grigg, Sailesh Kotecha

**Affiliations:** 1 Department of Child Health, School of Medicine, Cardiff University, Cardiff, United Kingdom; 2 Centre for Genomics and Child Health, Queen Mary University of London, London, United Kingdom; Australian National University, AUSTRALIA

## Abstract

**Background:**

Many but not all studies suggest an association between air pollution exposure and infant mortality. We sought to investigate whether pollution exposure is differentially associated with all-cause neonatal or postneonatal mortality, or specific causes of infant mortality.

**Methods and findings:**

We separately investigated the associations of exposure to particulate matter with aerodynamic diameter ≤ 10 μm (PM_10_), nitrogen dioxide (NO_2_), and sulphur dioxide (SO_2_) with all-cause infant, neonatal, and postneonatal mortality, and with specific causes of infant deaths in 7,984,366 live births between 2001 and 2012 in England and Wales. Overall, 51.3% of the live births were male, and there were 36,485 infant deaths (25,110 neonatal deaths and 11,375 postneonatal deaths). We adjusted for the following major confounders: deprivation, birthweight, maternal age, sex, and multiple birth. Adjusted odds ratios (95% CI; *p*-value) for infant deaths were significantly increased for NO_2_, PM_10_, and SO_2_ (1.066 [1.027, 1.107; *p* = 0.001], 1.044 [1.007, 1.082; *p* = 0.017], and 1.190 [1.146, 1.235; *p* < 0.001], respectively) when highest and lowest pollutant quintiles were compared; however, neonatal mortality was significantly associated with SO_2_ (1.207 [1.154, 1.262; *p* < 0.001]) but not significantly associated with NO_2_ and PM_10_ (1.044 [0.998, 1.092; *p* = 0.059] and 1.008 [0.966, 1.052; *p* = 0.702], respectively). Postneonatal mortality was significantly associated with all pollutants: NO_2_, 1.108 (1.038, 1.182; *p* < 0.001); PM_10_, 1.117 (1.050, 1.188; *p* < 0.001); and SO_2_, 1.147 (1.076, 1.224; *p* < 0.001). Whilst all were similarly associated with endocrine causes of infant deaths (NO_2_, 2.167 [1.539, 3.052; *p* < 0.001]; PM_10_, 1.433 [1.066, 1.926; *p* = 0.017]; and SO_2_, 1.558 [1.147, 2.116; *p* = 0.005]), they were differentially associated with other specific causes: NO_2_ and PM_10_ were associated with an increase in infant deaths from congenital malformations of the nervous (NO_2_, 1.525 [1.179, 1.974; *p* = 0.001]; PM_10_, 1.457 [1.150, 1.846; *p* = 0.002]) and gastrointestinal systems (NO_2_, 1.214 [1.006, 1.466; *p* = 0.043]; PM_10_, 1.312 [1.096, 1.571; *p* = 0.003]), and NO_2_ was also associated with deaths from malformations of the respiratory system (1.306 [1.019, 1.675; *p* = 0.035]). In contrast, SO_2_ was associated with an increase in infant deaths from perinatal causes (1.214 [1.156, 1.275; *p* < 0.001]) and from malformations of the circulatory system (1.172 [1.011, 1.358; *p* = 0.035]). A limitation of this study was that we were not able to study associations of air pollution exposure and infant mortality during the different trimesters of pregnancy. In addition, we were not able to control for all confounding factors such as maternal smoking.

**Conclusions:**

In this study, we found that NO_2_, PM_10_, and SO_2_ were differentially associated with all-cause mortality and with specific causes of infant, neonatal, and postneonatal mortality.

## Abstract 2

Sarah Kotecha and colleagues explore key air pollutants and associated deaths in newborns and infants.

## Author summary

**Why was this study done?:**

- Worldwide, in 2017, air pollution was thought to be responsible for an estimated 600,000 deaths of children aged under 5 years.
- Some, but not all, studies suggest an association between air pollution exposure and deaths during the first year of life.
- We investigated whether air pollution is associated with infant deaths after taking into account important factors such as deprivation, and also investigated which causes of death may be increased as a result of air pollution exposure.

**What did the researchers do and find?:**

- We used data for 7,984,366 babies born alive between 2001 and 2012 in England and Wales.
- We associated 3 air pollutants, namely particulate matter with aerodynamic diameter ≤ 10 μm (PM_10_), nitrogen dioxide (NO_2_), and sulphur dioxide (SO_2_), with all-cause infant deaths (deaths in the first year of life), neonatal deaths (deaths occurring within the first 28 days of life), and postneonatal deaths (deaths occurring from 28 days up to 1 year of age), and with specific causes of infant deaths.
- Infant and postneonatal deaths were significantly associated with increased exposure to NO_2_, PM_10_, and SO_2_; however, risk of neonatal death was only significantly associated with SO_2_.
- The 3 air pollutants seemed to be associated with different underlying causes of deaths.

**What do these findings mean?:**

- We have shown that the 3 air pollutants are associated in different ways with neonatal and postneonatal deaths, with all 3 associated with infant deaths but only SO_2_ associated with neonatal deaths, suggesting that SO_2_ exposure is more likely to act via the mother.
- We also show that air pollutants are associated with deaths via different specific causes, thus suggesting that each acts in a unique way.
- Only by understanding how air pollutants act to cause increased infant mortality can prevention therapies be developed to prevent both fetal and newborn mortality.

## Introduction

In 2017, the World Health Organization estimated that worldwide air pollution was responsible for 600,000 deaths in children under 5 years every year [1]. A systematic review reported an inconsistent link between particulate matter with aerodynamic diameter ≤ 10 μm (PM_10_) and infant mortality, although PM_10_ appeared to be associated with postneonatal mortality but not with neonatal mortality [2]. In a US study of approximately 4 million infants, it was reported that postneonatal mortality due to respiratory causes was associated with PM_10_ [3]. Infants with a high exposure to PM_10_ who were born with a normal birthweight were 45% more likely to die from respiratory causes when compared to infants with a low exposure. After adjustments for maternal education and other confounding variables, the associations markedly decreased, but a 10% higher risk of postneonatal death for the high exposure group compared to the low exposure group still remained [3]. In another study, for PM_10_ levels above 12 μg/m^3^ in 23 US metropolitan areas, the estimated proportion of all-cause mortality, sudden infant death syndrome (SIDS), and respiratory causes for postneonatal mortality were 6%, 16%, and 24%, respectively [4]. Similar associations have been noted between infant mortality and other pollutants including PM_2.5_, nitrogen dioxide (NO_2_), and sulphur dioxide (SO_2_) [5–9]. However, the studies thus far have not been able to clearly identify whether ambient pollutant exposures are associated with more neonatal deaths than postneonatal deaths, as it is very likely that the former may be associated with maternal exposures and the latter associated with direct exposure of the infant.

A number of studies, including a literature review, suggest possible associations between pollution exposure and causes of infant deaths, especially SIDS, but associations with other neonatal and postneonatal causes of death are less clear [10–13]. In particular, it is unclear whether the different pollutants are associated with differential causes of infant deaths after exposure. Since increased neonatal deaths attributable to pollution exposure may be via maternal exposures, and postneonatal mortality may be via direct exposure of the infant, we (a) investigated the association between the pollution exposures PM_10_, NO_2_, and SO_2_ and all-cause infant, neonatal, and postneonatal mortality separately and (b) identified the specific causes related to exposure to PM_10_, NO_2_, and SO_2_, in nearly 8 million live births between 2001 and 2012 in England and Wales, including after full adjustments for important confounders including deprivation.

## Methods

Mean annual values for NO_2_ and PM_10_ exposure for the years 2001 to 2012 and for SO_2_ exposure for the years 2002 to 2012 were obtained from the UK’s Department for Environment, Food and Rural Affairs (DEFRA). The data are collected by the UK government as part of the EU’s Air Quality Directive, and are collected directly from monitors throughout the UK and by verified modelling at a resolution of 1 km × 1 km, as described in detail by DEFRA [14]. Anonymised data for England and Wales for live births and deaths in the first year of life as well as additional demographic data, including deprivation score, sex, maternal age, birthweight, and singleton/multiple births, were obtained from the UK’s Office for National Statistics (ONS) for the years 2001 to 2012. Ethical approval was not required. Infant deaths (death occurring in the first year of life) were classified as neonatal deaths (death occurring within the first 28 days of life, i.e., at age 0–27 days) or postneonatal deaths (death occurring from 28 days to 1 year of age). Deprivation was reported by the English and Welsh Index of Multiple Deprivation (IMD) scores [15], which are measures of deprivation based on 7 domains of deprivation in England and 8 in Wales including wealth, schooling, and home ownership. Scores between England and Wales have been shown to be comparable [16]. These scores are calculated for all Lower Layer Super Output Areas (LSOAs) in England and Wales. LSOAs are small areas of similar population size, with an average of approximately 1,500 residents, or 650 households. There are 32,844 LSOAs in England and 1,909 in Wales. Each LSOA was also supplied with *x* and *y* coordinates, on the same scale as the DEFRA 1 km by 1 km grid, for its centre of mass—based on population distribution within the LSOA. The pollutant level for each combination of LSOA and year was allocated by determining the closest, i.e., minimum Euclidean distance, of the DEFRA grid points to each LSOA’s centre of mass. For each pollutant then, the levels for each combination of LSOA and year were ranked and banded into quintiles. By taking this approach, the quintiles as measures of pollutant represent the same range of actual pollutant levels over the whole time period. For each birth, the pollutant quintile was that for the LSOA and year of birth.

The differential association of each pollutant with all-cause infant, neonatal, and postneonatal mortality was tested individually (for the analyses of postneonatal deaths, the neonatal deaths were removed from the population at risk), through univariable and then multivariable regression with adjustment first for deprivation, and then for a combination of deprivation score, birthweight, maternal age, sex, and multiple birth. The association between pollutants and specific causes of infant death were similarly analysed. Level 1, then separately level 2, International Classification of Diseases–10th Revision (ICD-10) codes were tested through regression, first unadjusted then fully adjusted for deprivation, birthweight, maternal age, sex, and multiple birth. No adjustments were made for multiple testing. A *p*-value < 0.05 was considered significant. All analyses were performed using SPSS version 25. The analysis plan (S1 Text) was conceived prior to obtaining the data from ONS and DEFRA to assess the associations between air pollutants and neonatal, postneonatal, and infant deaths after adjustments were made for important confounders. In addition, we had aimed to associate the air pollutants with specific causes of neonatal deaths but were unable to do so due to the limited number of neonatal deaths. This study is reported as per the Strengthening the Reporting of Observational Studies in Epidemiology (STROBE) guideline (S1 STROBE Checklist).

## Results

**Fig 1:**
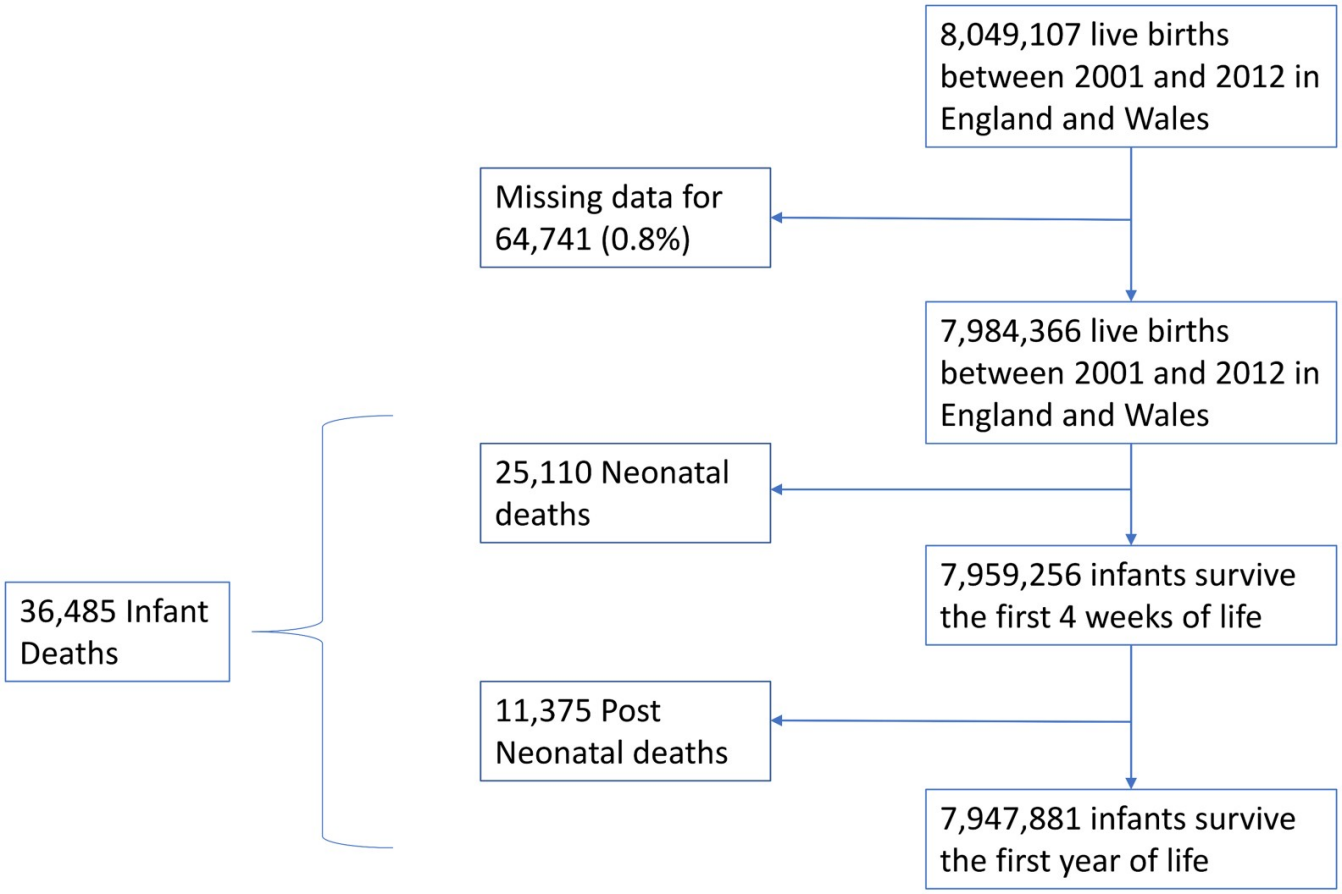
Flow chart of the included infants. PM_10_, particulate matter with aerodynamic diameter ≤ 10 μm.

Data were obtained for 8,049,107 live births in England and Wales between 1 January 2001 and 31 December 2012. There were complete data for all the variables for 7,984,366 live births (99.2%; data were missing for 64,741 [0.8%] live births). Of the included 7,984,366 live births, there were 36,485 (0.46%) infant deaths, consisting of 25,110 neonatal deaths (69% of infant deaths) and 11,375 postneonatal deaths (31% of infant deaths) (Fig 1). Demographics are shown in Table 1. Overall, more male infants died than female infants in all the death categories. More deaths occurred in infants born to mothers aged <20 years at the time of birth than mothers aged 21–30 years or those >30 years of age, for all death categories. As previously reported, there was a gradient of increased deaths with increasing deprivation for all death categories. More deaths occurred in multiple births than in singleton births in all the death categories.

**Table 1:** Demographics of the included population of live births, infant deaths, neonatal deaths, and postneonatal deaths.

Characteristic	Live births	Infant deaths	Neonatal deaths	Postneonatal deaths
**Total**	7,984,366 (100%)	36,485	25,110	11,375
**Sex**				
Female	3,891,228 (48.7%)	15,935 (43.7%)	11,031 (43.9%)	4,904 (43.1%)
Male	4,093138 (51.3%)	20,550 (56.3%)	14,079 (56.1%)	6,471 (56.9%)
**Maternal age**				
≤20 years	757,311 (9.5%)	4,874 (13.4%)	3,039 (12.1%)	1,835 (16.1%)
21–30 years	3,862,269 (48.4%)	17,558 (48.2%)	11,950 (47.6%)	5,608 (49.3%)
>30 years	3,364,786 (42.1%)	14,053 (38.5%)	10,121 (40.3%)	3,932 (34.6%)
**Quintile of deprivation**				
Least deprived	1,280,805 (16.0%)	3,977 (10.9%)	2,908 (11.6%)	1,069 (9.4%)
Second least deprived	1,318,948 (16.5%)	4,578 (12.5%)	3,315 (13.2%)	1,263 (11.1%)
Average deprived	1,460,888 (18.3%)	5,866 (16.1%)	4,134 (16.5%)	1,732 (15.2%)
Second most deprived	1,737,906 (21.8%)	8,486 (23.3%)	5,839 (23.2%)	2,647 (23.3%)
Most deprived	2,185,819 (27.4%)	13,578 (37.2%)	8,914 (35.5%)	4,664 (41.0%)
**Birthweight band**				
≥2,500 g	7,398,402 (92.7%)	12,843 (35.2%)	6,578 (26.2%)	6,265 (55.1%)
1,500–2,499 g	489,463 (6.1%)	5,603 (15.4%)	3,375 (13.4%)	2,228 (19.6%)
<1,500 g	96,501 (1.2%)	18,039 (49.4%)	15,157 (60.4%)	2,882 (25.3%)
**Multiple birth**				
Singleton birth	7,744,606 (97.0%)	31,528 (86.4%)	21,150 (84.2%)	10,378 (91.2%)
Multiple birth	239,760 (3.0%)	4,957 (13.6%)	3,960 (15.8%)	997 (8.8%)

Values are total number (percentage). Percentages in the live births column are related to total number of live births; percentages in the infant, neonatal, and postneonatal deaths columns are related to the total numbers shown the second row as denominator.

PM_10_, particulate matter with aerodynamic diameter ≤ 10 μm.

Annual geographical exposures for each pollutant were obtained from 2001 to 2012 for PM_10_ and NO_2_, and from 2002 to 2012 for SO_2_ and linked to each LSOA in England and Wales for each year (417,036 LSOAs for PM_10_, 416,671 for NO_2_, and 382,283 for SO_2_). Due to the non-linear nature of pollutant exposure, the exposures were categorised into quintiles rather than using linear modelling (S1 Table).

**Fig 2:**
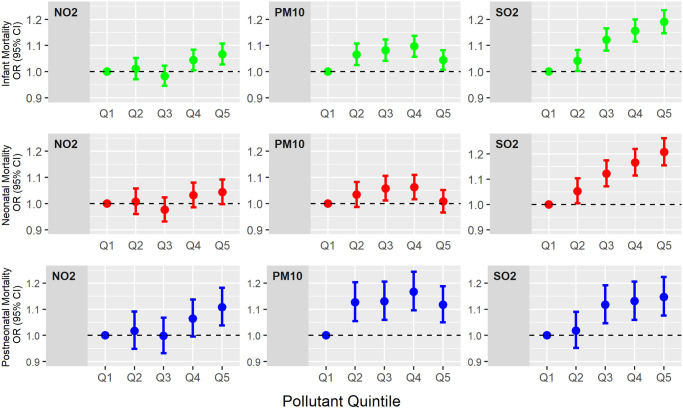
Differential associations of air pollutants with deaths. Adjusted odds ratios (ORs) and 95% CIs are shown. PM_10_, particulate matter with aerodynamic diameter ≤ 10 μm.

The absolute numbers of all-cause infant, neonatal, and postneonatal deaths are shown in S2 Table. In general, more births and infant deaths occurred in the highest quintile of exposure. When the lowest exposure quintile (quintile 1) was used as the reference value, increased exposure to all 3 pollutants was associated with markedly increased deaths in all cause mortality categories, with the highest exposure quintile (quintile 5) having an odds ratio (OR) (95% CI; *p*-value), of 1.240 (1.199, 1.282; *p* < 0.001) to 1.430 (1.382, 1.479; *p* < 0.001) for infant deaths, 1.208 (1.160, 1.257; *p* < 0.001) to 1.384 (1.327, 1.443; *p* < 0.001) for neonatal deaths, and 1.315 (1.237, 1.397; *p* < 0.001) to 1.542 (1.449, 1.640; *p* < 0.001) for postneonatal deaths (Table 2). The univariable ORs decreased markedly after adjustment for deprivation and in the fully adjusted model, which included deprivation, birthweight, maternal age, sex, and multiple birth, each of which independently had an association with infant mortality as shown in Table 1. In the fully adjusted model, the ORs for infant deaths were significantly increased: OR (95% CI; *p*-value) 1.066 (1.027, 1.107; *p* = 0.001), 1.044 (1.007, 1.082; *p* = 0.017), 1.190 (1.146, 1.235; *p* < 0.001) for NO_2_, PM_10_, and SO_2_, respectively, when quintile 5 was compared with quintile 1. Interestingly, however, the models resulted in differential associations with neonatal mortality rates: Only SO_2_ was significantly associated with increased neonatal deaths (1.207 [1.154, 1.262; *p* < 0.001]), with no significant association noted for NO_2_ (1.044 [0.998, 1.092; *p* = 0.059]) or PM_10_ (1.008 [0.966, 1.052; *p* = 0.702]) (Table 2). In contrast, all 3 were associated with postneonatal deaths when the first and fifth quintiles of exposure were compared (1.108 [1.038, 1.182; *p* < 0.001], 1.117 [1.050, 1.188; *p* < 0.001], 1.147 [1.076, 1.224; *p* < 0.001], respectively, for NO_2_, PM_10_, and SO_2_), albeit with different patterns of association for the different exposure quintiles, with PM_10_ having an association at all exposure quintiles, NO_2_ only at the highest quintile, and SO_2_ at quintile 3 and higher (Table 2; Fig 2).

**Table 2:** Differential associations of pollutants with deaths.

Pollutant and exposure quintile	Infant deaths	Neonatal deaths	Postneonatal deaths
**NO**_**2**_	*n* = 36,485	*n* = 25,110	*n* = 11,375
**Quintile 1**	1	1	1
** Quintile 2**	**1.070 (1.030, 1.111; *p* < 0.001)**	**1.059 (1.012, 1.108; *p* = 0.012)**	**1.094 (1.021, 1.172; *p* = 0.011)**
Adjusted for IMD	1.027 (0.988, 1.067; *p* = 0.164)	1.025 (0.980, 1.073; *p* = 0.270)	1.030 (0.961, 1.104; *p* = 0.396)
Fully adjusted	1.011 (0.971, 1.052; *p* = 0.579)	1.007 (0.960, 1.057; *p* = 0.747)	1.017 (0.949, 1.091; *p* = 0.618)
** Quintile 3**	**1.123 (1.083, 1.166; *p* < 0.001)**	**1.103 (1.056, 1.153; *p* < 0.001)**	**1.170 (1.094, 1.252; *p* < 0.001)**
Adjusted for IMD	1.020 (0.983, 1.059; *p* = 0.284)	1.020 (0.976, 1.067; *p* = 0.367)	1.020 (0.953, 1.091; *p* = 0.563)
Fully adjusted	0.983 (0.946, 1.023; *p* = 0.413)	0.976 (0.931, 1.023; *p* = 0.322)	0.998 (0.932, 1.068; *p* = 0.958)
** Quintile 4**	**1.281 (1.236, 1.327; *p* < 0.001)**	**1.244 (1.192, 1.298; *p* < 0.001)**	**1.368 (1.283, 1.459; *p* < 0.001)**
Adjusted for IMD	**1.090 (1.051, 1.130; *p* < 0.001)**	**1.089 (1.043, 1.138; *p* < 0.001)**	**1.091 (1.021, 1.165; *p* = 0.009)**
Fully adjusted	**1.044 (1.005, 1.084; *p* = 0.025)**	1.032 (0.986, 1.080; *p* = 0.166)	1.064 (0.996, 1.137; *p* = 0.063)
** Quintile 5**	**1.430 (1.382, 1.479; *p* < 0.001)**	**1.381 (1.326, 1.439; *p* < 0.001)**	**1.542 (1.449, 1.640; *p* < 0.001)**
Adjusted for IMD	**1.148 (1.108, 1.190; *p* < 0.001)**	**1.153 (1.105, 1.203; *p* < 0.001)**	**1.138 (1.068, 1.214; *p* < 0.001)**
Fully adjusted	**1.066 (1.027, 1.107; *p* = 0.001)**	1.044 (0.998, 1.092; *p* = 0.059)	**1.108 (1.038, 1.182; *p* < 0.005)**
**PM**_**10**_	*n* = 36,485	*n* = 25,110	*n* = 11,375
** Quintile 1**	1	1	1
** Quintile 2**	**1.096 (1.057, 1.136; *p* < 0.001)**	**1.069 (1.023, 1.117; *p* = 0.003)**	**1.158 (1.085, 1.237; *p* < 0.001)**
Adjusted for IMD	**1.085 (1.046, 1.125; *p* < 0.001)**	**1.061 (1.015, 1.108; *p* = 0.008)**	**1.141 (1.068, 1.218; *p* < 0.001)**
Fully adjusted	**1.065 (1.025, 1.107; *p* = 0.001)**	1.034 (0.987, 1.082; *p* = 0.151)	**1.127 (1.055, 1.204; *p* < 0.001)**
** Quintile 3**	**1.131 (1.091, 1.172; *p* < 0.001)**	**1.111 (1.064, 1.159; *p* < 0.001)**	**1.178 (1.104, 1.257; *p* < 0.001)**
Adjusted for IMD	**1.113 (1.074, 1.154; *p* < 0.001)**	**1.097 (1.051, 1.145; *p* < 0.001)**	**1.151 (1.079, 1.228; *p* < 0.001)**
Fully adjusted	**1.082 (1.042, 1.123; *p* < 0.001)**	**1.057 (1.011, 1.106; *p* = 0.015)**	**1.130 (1.059, 1.206; *p* < 0.001)**
** Quintile 4**	**1.207 (1.166, 1.249; *p* < 0.001)**	**1.182 (1.134, 1.232; *p* < 0.001)**	**1.264 (1.188, 1.346; *p* < 0.001)**
Adjusted for IMD	**1.163 (1.123, 1.204; *p* < 0.001)**	**1.146 (1.099, 1.195; *p* < 0.001)**	**1.202 (1.129, 1.280; *p* < 0.001)**
Fully adjusted	**1.097 (1.057, 1.137; *p* < 0.001)**	**1.062 (1.016, 1.109; *p* = 0.007)**	**1.167 (1.096, 1.243; *p* < 0.001)**
** Quintile 5**	**1.240 (1.199, 1.282; *p* < 0.001)**	**1.208 (1.160, 1.257; *p* < 0.001)**	**1.315 (1.237, 1.397; *p* < 0.001)**
Adjusted for IMD	**1.117 (1.080, 1.155; *p* < 0.001)**	**1.106 (1.062, 1.152; *p* < 0.001)**	**1.143 (1.075, 1.215; *p* < 0.001)**
Fully adjusted	**1.044 (1.007, 1.082; *p* = 0.017)**	1.008 (0.966, 1.052; *p* = 0.702)	**1.117 (1.050, 1.188; *p* < 0.001)**
**SO**_**2**_	*n* = 33,404	*n* = 23,011	*n* = 10,393
** Quintile 1**	1	1	1
** Quintile 2**	**1.067 (1.028, 1.108; *p* = 0.001)**	**1.068 (1.022, 1.117; *p* = 0.003)**	1.065 (0.995, 1.139; *p* = 0.066)
Adjusted for IMD	**1.045 (1.007, 1.085; *p* = 0.019)**	**1.051 (1.005, 1.099; *p* = 0.028)**	1.032 (0.965, 1.104; *p* = 0.351)
Fully adjusted	**1.041 (1.002, 1.083; *p* < 0.039)**	**1.052 (1.005, 1.103; *p* = 0.030)**	1.018 (0.952, 1.090; *p* = 0.588)
** Quintile 3**	**1.205 (1.162, 1.249; *p* < 0.001)**	**1.187 (1.137, 1.239; *p* < 0.001)**	**1.246 (1.168, 1.328; *p* < 0.001)**
Adjusted for IMD	**1.129 (1.089, 1.171; *p* < 0.001)**	**1.126 (1.078, 1.176; *p* < 0.001)**	**1.137 (1.065, 1.213; *p* < 0.001)**
Fully adjusted	**1.122 (1.080, 1.165; *p* < 0.001)**	**1.122 (1.072, 1.174; *p* < 0.001)**	**1.117 (1.046, 1.192; *p* < 0.005)**
** Quintile 4**	**1.318 (1.273, 1.366; *p* < 0.001)**	**1.297 (1.243, 1.353; *p* < 0.001)**	**1.366 (1.283, 1.455; *p* < 0.001)**
Adjusted for IMD	**1.178 (1.137, 1.221; *p* < 0.001)**	**1.182 (1.133, 1.234; *p* < 0.001)**	**1.168 (1.096, 1.245; *p* < 0.001)**
Fully adjusted	**1.156 (1.114, 1.200; *p* < 0.001)**	**1.165 (1.114, 1.219; *p* < 0.001)**	**1.131 (1.060, 1.206; *p* < 0.001)**
** Quintile 5**	**1.398 (1.350, 1.448; *p* < 0.001)**	**1.384 (1.327, 1.443; *p* < 0.001)**	**1.429 (1.342, 1.522; *p* < 0.001)**
Adjusted for IMD	**1.236 (1.193, 1.280; *p* < 0.001)**	**1.250 (1.198, 1.304; *p* < 0.001)**	**1.205 (1.130, 1.284; *p* < 0.001)**
Fully adjusted	**1.190 (1.146, 1.235; *p* < 0.001)**	**1.207 (1.154, 1.262; *p* < 0.001)**	**1.147 (1.076, 1.224; *p* < 0.001)**

Data are given as odds ratio (95% CI; *p*-value). Fully adjusted is adjusted for Index of Multiple Deprivation (IMD), birthweight, maternal age, sex, and multiple birth. Significant differences shown in bold.

PM_10_, particulate matter with aerodynamic diameter ≤ 10 μm.

We looked at each pollutant separately and for each pollutant estimated the extra number of infant deaths due to that pollutant after adjustment for the confounders deprivation, birthweight, maternal age, infant sex, and multiple birth when comparing quintiles 2–5 to the lowest quintile of pollution. There were estimated numbers of extra deaths of 62, 157, and 261 each year from exposure to NO_2_, PM_10_, and SO_2_, respectively. Caution is required with interpreting these figures as the 3 pollutants may occur simultaneously and other unavailable factors such as maternal smoking may be important.

We next investigated the specific causes of infant mortality against pollution exposures, with the absolute number of deaths against pollution exposure quintiles shown in S3 Table, which shows increased rates in the highest quintile of exposure. Infant deaths from perinatal causes (*n* = 21,496) and congenital malformations (*n* = 6,325) predominated. Using the lowest quintile as the reference value, for NO_2_, after full adjustment, infant deaths from congenital malformations (OR [95% CI; *p*-value] 1.159 [1.063, 1.264; *p* = 0.001]), endocrine disorders (2.167 [1.539, 3.052; *p* < 0.001]), and blood disorders (3.197 [1.472, 6.941; *p* = 0.003]) were strongly associated with the highest exposure quintile, as well as perinatal causes to a lesser extent (1.060 [1.009, 1.114; *p* = 0.019]) (Table 3). For PM_10_, infant deaths from congenital malformations (1.121 [1.034, 1.217; *p* = 0.006]) and endocrine disorders (1.433 [1.066, 1.926; *p* = 0.017]) were associated with the highest exposure quintile, with relatively high non-significant ORs observed for infant deaths from respiratory (1.249 [0.973, 1.602; *p* = 0.081]) and blood disorders (1.923 [0.997, 3.709; *p* = 0.051]), although smaller numbers were available for study. Perinatal (1.214 [1.156, 1.275; *p* < 0.001]) and endocrine (1.558 [1.147, 2.116; *p* = 0.005]) disorders had the strongest associations with infant deaths in the highest quintile of SO_2_ exposure. We next investigated the relevant specific causes (Table 4). NO_2_ and PM_10_ exposure at the highest quintile were associated with increased infant deaths from congenital malformations of the nervous system (1.525 [1.179, 1.974; *p* = 0.001] and 1.457 [1.150, 1.846; *p* = 0.002], respectively) and the gastrointestinal system (1.214 [1.006, 1.466; *p* = 0.043] and 1.312 [1.096, 1.571; *p* = 0.003], respectively), but NO_2_ exposure at the highest quintile was also associated with increased infant deaths from congenital malformations of the respiratory system (1.306 [1.019, 1.675; *p* = 0.035]), metabolic disorders (2.025 [1.427, 2.873; *p* < 0.001]), and ‘other’ bacterial diseases (1.371 [1.018, 1.846; *p* = 0.038]). A different pattern for causes of infant deaths was seen for the highest exposure to SO_2_, with significant associations for congenital malformations of the circulatory system (1.172 [1.011, 1.358; *p* = 0.035]) and perinatal cardiorespiratory causes (1.351 [1.240, 1.471; *p* < 0.001]) but with associations similar to those of NO_2_ for metabolic disorders (1.455 [1.064, 1.990; *p* = 0.019]) and ‘other’ bacterial diseases (1.738 [1.277, 2.366; *p* < 0.001]).

**Table 3:** Association between specific causes of infant deaths and pollutants.

Pollutant and exposure quintile	Perinatal	Congenital malformations	Respiratory	Endocrine	Neoplasm	Blood
**NO**_**2**_	*n* = 21,496	*n* = 6,325	*n* = 792	*n* = 547	*n* = 212	*n* = 111
**Quintile 1**	**1**	**1**	**1**	**1**	**1**	**1**
**Quintile 2**	**1.067 (1.016, 1.120; *p* = 0.009)**	1.050 (0.956, 1.153: *p* = 0.306)	1.121 (0.857, 1.466; *p* = 0.403)	1.340 (0.912, 1.967; *p* = 0.135)	0.997 (0.633, 1.570; *p* = 0.992)	1.527 (0.640, 3.641; *p* = 0.339)
Adjusted	1.017 (0.965, 1.072; *p* = 0.512)	0.995 (0.905, 1.093; *p* = 0.918)	0.989 (0.756, 1.295; *p* = 0.9410	1.251 (0.851, 1.839; *p* = 0.253)	0.950 (0.603, 1.498; *p* = 0.828)	1.511 (0.632, 3.607; *p* = 0.353)
**Quintile 3**	**1.100 (1.049, 1.155; *p* < 0.001)**	**1.133 (1.034, 1.240; *p* = 0.007)**	**1.355 (1.050, 1.747; *p* = 0.019)**	**1.913 (1.339, 2.734; *p* < 0.001)**	0.954 (0.607, 1.498; *p* = 0.840)	1.527 (0.647, 3.603; *p* = 0.333)
Adjusted	0.975 (0.926, 1.026; *p* = 0.337)	0.993 (0.906, 1.088; *p* = 0.887)	1.058 (0.817, 1.368; *p* = 0.667)	**1.656 (1.156, 2.373; *p* = 0.006)**	0.881 (0.558, 1.389; *p* = 0.586)	1.441 (0.608, 3.414; *p* = 0.406)
**Quintile 4**	**1.234 (1.178, 1.292; *p* < 0.001)**	**1.385 (1.271, 1.509; *p* < 0.001)**	**1.560 (1.222, 1.991; *p* < 0.001)**	**2.481 (1.765, 3.488; *p* < 0.001)**	0.952 (0.612, 1.481; *p* = 0.830)	**2.686 (1.228, 5.875; *p* = 0.013)**
Adjusted	1.031 (0.980, 1.084; *p* = 0.227)	**1.112 (1.019, 1.215; *p* = 0.017)**	1.068 (0.831, 1.373; *p* = 0.604)	**1.965 (1.388, 2.781; *p* < 0.001)**	0.850 (0.540, 1.337; *p* = 0.482)	**2.413 (1.090, 5.344; *p* = 0.030)**
**Quintile 5**	**1.398 (1.338, 1.461; *p* < 0.001)**	**1.572 (1.448, 1.707; *p* < 0.001)**	**1.493 (1.173, 1.901; *p* = 0.001)**	**2.935 (2.109, 4.084; *p* < 0.001)**	0.952 (0.619, 1.465; *p* = 0.826)	**3.678 (1.734, 7.803; *p* = 0.001)**
Adjusted	**1.060 (1.009, 1.114; *p* = 0.019)**	**1.159 (1.063, 1.264; *p* = 0.001)**	0.917 (0.713, 1.179; *p* = 0.500)	**2.167 (1.539, 3.052; *p* < 0.001)**	0.824 (0.524, 1.293; *p* = 0.400)	**3.197 (1.472, 6.941; *p* = 0.003)**
**PM**_**10**_	*n* = 21,496	*n* = 6,325	*n* = 792	*n* = 547	*n* = 212	*n* = 111
**Quintile 1**	**1**	**1**	**1**	**1**	**1**	**1**
**Quintile 2**	**1.069 (1.019, 1.121; *p* = 0.006)**	**1.147 (1.051, 1.252; *p* = 0.002)**	**1.558 (1.203, 2.017; *p* = 0.001)**	**1.548 (1.135, 2.112; *p* = 0.006)**	1.282 (0.809, 2.029; *p* = 0.290)	1.053 (0.487, 2.278; *p* = 0.895)
Adjusted	1.030 (0.979, 1.083; *p* = 0.251)	**1.115 (1.022, 1.217; *p* = 0.014)**	**1.495 (1.154, 1.936; *p* = 0.002)**	**1.498 (1.098, 2.044; *p* = 0.011)**	1.266 (0.799, 2.005; *p* = 0.315)	1.047 (0.484, 2.265; *p* = 0.906)
**Quintile 3**	**1.114 (1.063, 1.167; *p* < 0.001)**	**1.154 (1.058, 1.257; *p* = 0.001)**	**1.456 (1.123, 1.886; *p* = 0.005)**	1.297 (0.943, 1.783; *p* = 0.110)	1.054 (0.656, 1.694; *p* = 0.827)	1.218 (0.582, 2.551; *p* = 0.600)
Adjusted	**1.057 (1.006, 1.111; *p* = 0.028)**	**1.100 (1.009, 1.200; *p* = 0.030)**	**1.372 (1.059, 1.778; *p* = 0.017)**	1.237 (0.900, 1.702; *p* = 0.190)	1.037 (0.645, 1.668; *p* = 0.879)	1.196 (0.571, 2.504; *p* = 0.636)
**Quintile 4**	**1.219 (1.166, 1.275; *p* < 0.001)**	**1.164 (1.070, 1.267; *p* < 0.001)**	**1.624 (1.264, 2.086; *p* < 0.001)**	1.593 (1.178, 2.155; *p* = 0.003)	1.173 (0.744, 1.849; *p* = 0.493)	2.042 (1.048, 3.975; *p* = 0.036)
Adjusted	**1.088 (1.037, 1.141; *p* = 0.001)**	1.065 (0.979, 1.160; *p* = 0.143)	**1.458 (1.134, 1.874; *p* = 0.003)**	**1.472 (1.088, 1.993; *p* = 0.012)**	1.138 (0.721, 1.795; *p* = 0.580)	1.946 (0.998, 3.794; *p* = 0.051)
**Quintile 5**	**1.240 (1.187, 1.295; *p* < 0.001)**	**1.332 (1.229, 1.444; *p* < 0.001)**	**1.548 (1.209, 1.982; *p* = 0.001)**	**1.696 (1.265, 2.273; *p* < 0.001)**	1.186 (0.761, 1.847; *p* = 0.451)	**2.139 (1.115, 4.102; *p* = 0.022)**
Adjusted	1.028 (0.981, 1.078; *p* = 0.249)	**1.121 (1.034, 1.217; *p* = 0.006)**	1.249 (0.973, 1.602; *p* = 0.081)	**1.433 (1.066, 1.926; *p* = 0.017)**	1.124 (0.719, 1.759; *p* = 0.608)	1.923 (0.997, 3.709; *p* = 0.051)
**SO**_**2**_	*n* = 19,695	*n* = 5,819	*n* = 717	*n* = 504	*n* = 199	*n* = 104
**Quintile 1**	**1**	**1**	**1**	**1**	**1**	**1**
**Quintile 2**	**1.081 (1.030, 1.134; *p* = 0.001)**	1.000 (0.915, 1.094; *p* = 0.997)	1.018 (0.783, 1.324; *p* = 0.893)	1.159 (0.829, 1.620; *p* = 0.388)	1.021 (0.666, 1.566; *p* = 0.924)	**0.466 (0.227, 0.955; *p* = 0.037)**
Adjusted	**1.074 (1.020, 1.130; *p* = 0.006)**	0.968 (0.885, 1.059; *p* = 0.476)	0.947 (0.728, 1.232; *p* = 0.684)	1.103 (0.789, 1.543; *p* = 0.566)	0.990 (0.645, 1.519; *p* = 0.963)	**0.447 (0.218, 0.918; *p* = 0.028)**
**Quintile 3**	**1.190 (1.136, 1.247; *p* < 0.001)**	**1.185 (1.088, 1.290; *p* < 0.001)**	1.226 (0.955, 1.574; *p* = 0.109)	**1.629 (1.194, 2.221; *p* = 0.002)**	0.941 (0.611, 1.451; *p* = 0.784)	0.696 (0.372, 1.302; *p* = 0.257)
Adjusted	**1.138 (1.083, 1.196; *p* < 0.001)**	1.076 (0.988, 1.173; *p* = 0.093)	1.034 (0.804, 1.329; *p* = 0.794)	**1.436 (1.051, 1.961; *p* = 0.023)**	0.887 (0.574, 1.371; *p* = 0.590)	0.627 (0.334, 1.179; *p* = 0.147)
**Quintile 4**	**1.282 (1.225, 1.342; *p* < 0.001)**	**1.384 (1.275, 1.503; *p* < 0.001)**	**1.538 (1.212, 1.951; *p* < 0.001)**	**1.892 (1.399, 2.558; *p* < 0.001)**	0.840 (0.539, 1.310; *p* = 0.443)	1.214 (0.705, 2.091; *p* = 0.483)
Adjusted	**1.169 (1.113, 1.228; *p* < 0.001)**	**1.174 (1.080, 1.277; *p* < 0.001)**	1.162 (0.912, 1.479; *p* = 0.225)	**1.532 (1.128, 2.080; *p* = 0.006)**	0.767 (0.489, 1.204; *p* = 0.249)	1.024 (0.589, 1.781; *p* = 0.933)
**Quintile 5**	**1.375 (1.314, 1.439; *p* < 0.001)**	**1.306 (1.201, 1.421; *p* < 0.001)**	**1.509 (1.187, 1.918; *p* = 0.001)**	**1.958 (1.449, 2.646; *p* < 0.001)**	0.863 (0.553, 1.346; *p* = 0.515)	0.956 (0.536, 1.704; *p* = 0.879)
Adjusted	**1.214 (1.156, 1.275; *p* < 0.001)**	1.085 (0.996, 1.182; *p* = 0.061)	1.100 (0.862, 1.404; *p* = 0.445)	**1.558 (1.147, 2.116; *p* = 0.005)**	0.780 (0.497, 1.226; *p* = 0.282)	0.792 (0.440, 1.427; *p* = 0.438)

Data are given as odds ratio (95% CI; *p*-value).

PM_10_, particulate matter with aerodynamic diameter ≤ 10 μm.

**Table 4:** Association between detailed specific causes of infant deaths and pollutants.

Pollutant and exposure quintile	Respiratory/cardiovascular disorders specific to perinatal period	Congenital malformations of the nervous system	Congential malformations of the gastrointestinal system	Congenital malformations of the circulatory system	Congenital malformations of the respiratory system	Metabolic disorders	Infections specific to the perinatal period	Influenza and pneumonia	Other bacterial diseases
**NO**_**2**_	*n* = 6,536	*n* = 767	*n* = 1,372	*n* = 2,123	*n* = 825	*n* = 498	*n* = 2,275	*n* = 364	*n* = 545
**Quintile 1**	1	1	1	1	1	1	1	1	1
**Quintile 2**	1.035 (0.949, 1.130) *p* = 0.434	1.067 (0.796, 1.431) *p* = 0.665	**1.273 (1.045, 1.551)** ***p* = 0.017**	1.037 (0.890, 1.208) *p* = 0.643	1.209 (0.923, 1.583) *p* = 0.168	1.289 (0.871, 1.907) *p* = 0.204	1.095 (0.947, 1.266) *p* = 0.223	1.086 (0.720, 1.638) *p* = 0.694	1.169 (0.847, 1.611) *p* = 0.342
Adjusted	1.003 (0.918, 1.095) *p* = 0.955	0.977 (0.727, 1.311) *p* = 0.875	1.211 (0.992, 1.478) *p* = 0.059	0.989 (0.848, 1.152) *p* = 0.885	1.147 (0.875, 1.504) *p* = 0.320	1.209 (0.817, 1.791) *p* = 0.343	1.051 (0.908, 1.216) *p* = 0.509	0.933 (0.617, 1.410) *p* = 0.741	1.088 (0.788, 1.502) *p* = 0.607
**Quintile 3**	1.019 (0.935, 1.111) *p* = 0.670	1.298 (0.983, 1.713) *p* = 0.066	1.080 (0.883, 1.321) *p* = 0.455	0.992 (0.852, 1.154) *p* = 0.914	**1.352 (1.042, 1.754)** ***p* = 0.023**	**1.727 (1.196, 2.492)** ***p* = 0.004**	1.126 (0.977, 1.299) *p* = 0.101	**1.471 (1.004, 2.154)** ***p* = 0.048**	1.266 (0.927, 1.729) *p* = 0.138
Adjusted	0.936 (0.857, 1.022) *p* = 0.138	1.071 (0.809, 1.418) *p* = 0.632	0.939 (0.766, 1.152) *p* = 0.547	0.885 (0.759, 1.031) *p* = 0.117	1.162 (0.893, 1.511) *p* = 0.263	**1.512 (1.045, 2.188)** ***p* = 0.028**	1.015 (0.879, 1.173) *p* = 0.836	1.084 (0.737, 1.595) *p* = 0.683	1.104 (0.806, 1.512) *p* = 0.539
**Quintile 4**	**1.170 (1.078, 1.27)** ***p* < 0.001**	**1.601 (1.23,2.083)** ***p* < 0.001**	**1.402 (1.161, 1.692)** ***p* < 0.001**	**1.224 (1.061, 1.411)** ***p* = 0.005**	**1.629 (1.271, 2.087)** ***p* < 0.001**	**2.347 (1.659, 3.32)** ***p* < 0.001**	**1.169 (1.018, 1.343)** ***p* = 0.027**	**1.663 (1.151, 2.403)** ***p* = 0.007**	1.345 (0.994, 1.821) *p* = 0.055
Adjusted	1.022 (0.939, 1.112) *p* = 0.619	1.185 (0.905, 1.551) *p* = 0.218	1.127 (0.928, 1.367) *p* = 0.227	1.012 (0.874, 1.171) *p* = 0.876	1.255 (0.974, 1.617) *p* = 0.079	**1.896 (1.331, 2.702)** ***p* < 0.001**	0.990 (0.859, 1.142) *p* = 0.892	1.048 (0.719, 1.528) *p* = 0.808	1.092 (0.801, 1.489) *p* = 0.577
**Quintile 5**	**1.317 (1.217, 1.424)** ***p* < 0.001**	**2.285 (1.785, 2.925)** ***p* < 0.001**	**1.694 (1.416, 2.026)** ***p* < 0.001**	**1.261 (1.098, 1.448)** ***p* = 0.001**	**1.847 (1.454, 2.346)** ***p* < 0.001**	**2.678 (1.911, 3.753)** ***p* < 0.001**	1.140 (0.995, 1.306) *p* = 0.059	**1.691 (1.179, 2.424)** ***p* = 0.004**	**1.751 (1.317, 2.328)** ***p* < 0.001**
Adjusted	1.067 (0.982, 1.160) *p* = 0.126	**1.525 (1.179, 1.974)** ***p* = 0.001**	**1.214 (1.006, 1.466)** ***p* = 0.043**	0.970 (0.839, 1.122) *p* = 0.680	**1.306 (1.019, 1.675)** ***p* = 0.035**	**2.025 (1.427, 2.873)** ***p* < 0.001**	0.892 (0.773, 1.029) *p* = 0.116	0.952 (0.655, 1.384) *p* = 0.796	**1.371 (1.018, 1.846)** ***p* = 0.038**
**PM**_**10**_	*n* = 6,536	*n* = 767	*n* = 1,372	*n* = 2,123	*n* = 825	*n* = 498	*n* = 2,275	*n* = 364	*n* = 545
**Quintile 1**	1	1	1	1	1	1	1	1	1
**Quintile 2**	1.007 (0.926, 1.096) *p* = 0.865	1.285 (0.991, 1.666) *p* = 0.059	1.152 (0.945, 1.405) *p* = 0.162	1.128 (0.976, 1.304) *p* = 0.103	1.127 (0.880, 1.443) *p* = 0.345	**1.406 (1.020, 1.940)** ***p* = 0.038**	0.998 (0.869, 1.146) *p* = 0.972	**1.652 (1.129, 2.417)** ***p* = 0.010**	1.129 (0.828, 1.539) *p* = 0.444
Adjusted	0.979 (0.900, 1.066) *p* = 0.629	1.235 (0.952, 1.601) *p* = 0.112	1.106 (0.906, 1.350) *p* = 0.324	1.105 (0.956, 1.277) *p* = 0.178	1.103 (0.861, 1.413) *p* = 0.437	1.364 (0.989, 1.882) *p* = 0.059	0.969 (0.844, 1.114) *p* = 0.660	**1.585 (1.083, 2.321)** ***p* = 0.018**	1.102 (0.808, 1.503) *p* = 0.540
**Quintile 3**	1.034 (0.952, 1.123) *p* = 0.431	1.188 (0.915, 1.543) *p* = 0.196	1.300 (1.073, 1.575) *p* = 0.007	1.072 (0.927, 1.239) *p* = 0.349	1.256 (0.989, 1.595) *p* = 0.062	1.269 (0.917, 1.756) *p* = 0.151	0.982 (0.856, 1.126) *p* = 0.792	**1.657 (1.136, 2.417)** ***p* = 0.009**	**1.398 (1.042, 1.875)** ***p* = 0.025**
Adjusted	0.991 (0.911, 1.077) *p* = 0.823	1.118 (0.861, 1.452) *p* = 0.403	1.225 (1.010, 1.485) *p* = 0.039	1.036 (0.896, 1.198) *p* = 0.634	1.203 (0.947, 1.529) *p* = 0.130	1.214 (0.877, 1.681) *p* = 0.242	0.940 (0.819, 1.079) *p* = 0.378	**1.562 (1.071, 2.278)** ***p* = 0.021**	**1.350 (1.007, 1.812)** ***p* = 0.045**
**Quintile 4**	1.093 (1.008, 1.184) *p* = 0.030	1.181 (0.914, 1.528) *p* = 0.204	1.385 (1.150, 1.669) *p* = 0.001	1.042 (0.903, 1.203) *p* = 0.569	1.210 (0.955, 1.534) *p* = 0.115	**1.477 (1.082, 2.016)** ***p* = 0.014**	1.047 (0.917, 1.196) *p* = 0.500	**1.600 (1.100, 2.326)** ***p* = 0.014**	1.317 (0.983, 1.764) *p* = 0.065
Adjusted	1.000 (0.922, 1.084) *p* = 0.991	1.053 (0.814, 1.362) *p* = 0.695	1.205 (0.999, 1.454) *p* = 0.051	0.977 (0.846, 1.127) *p* = 0.745	1.109 (0.874, 1.406) *p* = 0.395	**1.369 (1.003, 1.869)** ***p* = 0.048**	0.954 (0.835, 1.091) *p* = 0.494	1.432 (0.985, 2.083) *p* = 0.060	1.241 (0.926, 1.663) *p* = 0.149
**Quintile 5**	**1.142 (1.057, 1.234)** ***p* = 0.001**	**1.809 (1.432, 2.287)** ***p* < 0.001**	**1.627 (1.362, 1.943)** ***p* < 0.001**	1.137 (0.991, 1.304) *p* = 0.067	**1.430 (1.143, 1.791)** ***p* = 0.002**	**1.569 (1.161, 2.121)** ***p* = 0.003**	0.961 (0.842, 1.096) *p* = 0.550	1.455 (1.003, 2.111) *p* = 0.048	**1.407 (1.061, 1.866)** ***p* = 0.018**
Adjusted	0.989 (0.913, 1.070) *p* = 0.774	**1.457 (1.150, 1.846)** ***p* = 0.002**	**1.312 (1.096, 1.571)** ***p* = 0.003**	0.992 (0.864, 1.140) *p* = 0.912	1.195 (0.953, 1.500) *p* = 0.123	1.336 (0.986, 1.811) *p* = 0.062	0.830 (0.726, 0.948) *p* = 0.006	1.151 (0.791, 1.673) *p* = 0.462	1.276 (0.960, 1.697) *p* = 0.094
**SO**_**2**_	*n* = 5,904	*n* = 707	*n* = 1,290	*n* = 1,957	*n* = 759	*n* = 460	*n* = 2,114	*n* = 321	*n* = 489
**Quintile 1**	1	1	1	1	1	1	1	1	1
**Quintile 2**	**1.097 (1.003, 1.200)** ***p* = 0.043**	1.102 (0.849, 1.431) *p* = 0.466	1.059 (0.885, 1.268) *p* = 0.532	1.061 (0.910, 1.237) *p* = 0.451	0.833 (0.656, 1.058) *p* = 0.135	1.083 (0.769, 1.527) *p* = 0.647	1.021 (0.889, 1.172) *p* = 0.771	0.933 (0.623, 1.398) *p* = 0.736	1.103 (0.780, 1.561) *p* = 0.579
Adjusted	1.089 (0.995, 1.191) *p* = 0.065	1.040 (0.800, 1.351) *p* = 0.770	1.036 (0.864, 1.242) *p* = 0.701	1.033 (0.886, 1.205) *p* = 0.677	0.797 (0.628, 1.013) *p* = 0.064	1.034 (0.733, 1.458) *p* = 0.849	0.999 (0.869, 1.148) *p* = 0.984	0.850 (0.567, 1.275) *p* = 0.432	1.043 (0.737, 1.477) *p* = 0.812
**Quintile 3**	**1.300 (1.193, 1.416)** ***p* < 0.001**	**1.288 (1.002, 1.654)** ***p* = 0.048**	1.149 (0.964, 1.369) *p* = 0.122	**1.234 (1.065, 1.430)** ***p* = 0.005**	0.909 (0.721, 1.146) *p* = 0.419	**1.488 (1.083, 2.045)** ***p* = 0.014**	0.997 (0.868, 1.144) *p* = 0.962	1.157 (0.790, 1.694) *p* = 0.453	**1.836 (1.345, 2.506)** ***p* < 0.001**
Adjusted	**1.251 (1.147, 1.364)** ***p* < 0.001**	1.112 (0.864, 1.431) *p* = 0.409	1.060 (0.888, 1.266) *p* = 0.517	1.144 (0.987, 1.327) *p* = 0.075	0.802 (0.635, 1.013) *p* = 0.064	1.320 (0.959, 1.818) *p* = 0.089	0.936 (0.814, 1.076) *p* = 0.350	0.936 (0.638, 1.374) *p* = 0.736	**1.639 (1.198, 2.241)** ***p* = 0.002**
**Quintile 4**	**1.344 (1.235, 1.463)** ***p* < 0.001**	**1.651 (1.302, 2.095)** ***p* < 0.001**	1.128 (0.946, 1.345) *p* = 0.179	**1.352 (1.170, 1.562)** ***p* < 0.001**	1.148 (0.923, 1.428) *p* = 0.216	**1.712 (1.257, 2.333)** ***p* = 0.001**	1.100 (0.962, 1.259) *p* = 0.165	**1.688 (1.186, 2.401)** ***p* = 0.004**	**1.645 (1.198, 2.258)** ***p* = 0.002**
Adjusted	**1.246 (1.143, 1.359)** ***p* < 0.001**	**1.290 (1.013, 1.643)** ***p* = 0.039**	0.969 (0.810, 1.160) *p* = 0.732	**1.187 (1.025, 1.374)** ***p* = 0.022**	0.923 (0.739, 1.153) *p* = 0.480	**1.403 (1.025, 1.920)** ***p* = 0.0340**	0.980 (0.854, 1.124) *p* = 0.772	1.194 (0.835, 1.708) *p* = 0.331	1.355 (0.983, 1.867) *p* = 0.064
**Quintile 5**	**1.491 (1.372, 1.621)** ***p* < 0.001**	**1.335 (1.040, 1.713)** ***p* = 0.023**	1.112 (0.931, 1.328) *p* = 0.241	**1.358 (1.175, 1.569)** ***p* < 0.001**	1.094 (0.876, 1.366) *p* = 0.430	**1.804 (1.326, 2.455)** ***p* < 0.001**	1.069 (0.932, 1.225) *p* = 0.341	**1.613 (1.129, 2.306)** ***p* = 0.009**	**2.199 (1.624, 2.978)** ***p* < 0.001**
Adjusted	**1.351 (1.240, 1.471)** ***p* < 0.001**	1.009 (0.783, 1.300) *p* = 0.944	0.912 (0.761, 1.093) *p* = 0.318	**1.172 (1.011, 1.358)** ***p* = 0.035**	0.854 (0.681, 1.071) *p* = 0.171	**1.455 (1.064, 1.990)** ***p* = 0.019**	0.921 (0.801, 1.058) *p* = 0.244	1.092 (0.759, 1.571) *p* = 0.634	**1.738 (1.277, 2.366)** ***p* < 0.001**

Data are given as odds ratio (95% CI) *p*-value.

PM_10_, particulate matter with aerodynamic diameter ≤ 10 μm.

## Discussion

In this large population-based study of nearly 8 million live births, we have shown that the major ambient pollutants, NO_2_, PM_10_, and SO_2_, were associated with infant mortality as suggested by several previous studies [3,5–9]. We extended our univariable observations by accounting for major determinants of infant mortality including deprivation, birthweight, sex, multiple birth, and age of the mother. Additionally, since pollutants may act either via the mother or directly on the infant, we separately studied the associations of the pollutants with neonatal and postneonatal deaths. We observed that all 3 exposures were associated with both neonatal and postneonatal mortality when univariable analyses were conducted, but after full adjustment, only SO_2_ was strongly associated with neonatal mortality even at low exposures, whilst NO_2_ and PM_10_ appeared to influence neonatal mortality to a far lesser extent. In contrast, after full adjustment, all 3 were associated with postneonatal mortality, although NO_2_ only at the highest exposure, PM_10_ even at low exposure, and SO_2_ at intermediate and high exposure. Most interestingly, we noted that the 3 exposures had differential associations with specific causes of infant mortality. SO_2_ had the greatest association with perinatal causes, in keeping with its greater association with neonatal deaths, but all 3 were associated with mortality from congenital malformations in different ways: NO_2_ and PM_10_ were both associated with increased infant deaths from congenital malformations of both the nervous and gastrointestinal systems, and NO_2_ was also associated with deaths from malformations of the respiratory system; in contrast, SO_2_ was associated with increased infant deaths from malformations of the circulatory system but not nervous or gastrointestinal systems. All 3, however, were associated with endocrine causes of infant deaths.

The majority of PM_10_ and NO_2_ is derived from road traffic, especially in urban areas, whereas the production of SO_2_ differs, with a large amount being produced by industry [17]. A recent review by Liu and Grigg noted that pollution levels in multiple areas of the UK often exceed EU legal limits and WHO guidelines on pollution levels [17]. Therefore, studying the human consequences of pollution exposure remains of great importance. In our study, the greater association of SO_2_ with neonatal mortality suggests that it may act via the mother, as newborns in the first month of life are less likely to be exposed to environmental pollutants. This observation was further strengthened by the association between SO_2_ and perinatal cardiorespiratory causes of infant deaths (Table 4). These data suggest that the fetus is likely affected by an as yet unknown mechanism, which may include transfer of 1 or more factors to the fetus via the maternal–placental circulation, accumulation in the placenta, or impacts on maternal health that subsequently impact on the fetus. Additional studies, most likely using animal models, may help identify the mechanisms of action (including inflammatory and oxidant pathways) of air pollutants on both fetuses and infants. Similar events were not observed with NO_2_ or PM_10_, thus suggesting that these 2 factors are less likely to act via the mother. How these differential associations occur and how they cause neonatal mortality will be of great interest. Pollution exposure in pregnancy has been investigated, with associations with stillbirths, preterm delivery, and low birthweight being reported [18–20]. In addition, a possible association between spontaneous abortions and premature birth and air pollutants has been reported [21]. Whether SO_2_ acts via direct means or indirectly via impacts on micronutrients or heavy metals is less clear [22].

All 3 pollutants were associated with postneonatal mortality, albeit with different patterns of association at different levels of exposure, with PM_10_ associated with postneonatal mortality even at low exposure, SO_2_ at intermediate and high exposure, and NO_2_ only at the highest exposure. It is very likely that these associations act via direct exposure of the infant. Animal models of pollutant exposures have shown that the pollutants most likely act via pulmonary inflammation. Kulkarni and colleagues showed that the induction of pulmonary inflammation may be mediated via uptake of carbon particles by alveolar macrophages [23]. Using an animal model, Carosino et al. have shown that PM_10_ promotes allergic pulmonary inflammation [24]. Li et al. have demonstrated that SO_2_ affects the airway inflammatory and immune responses of asthmatic rats and enhances their susceptibility by aggravating inflammatory responses in the lung [25]. Other pollutants such as ozone may cause oxidative stress, inflammatory responses, and immunologic dysfunction in animal models [26].

A recent review noted that causal evidence linking air pollution exposure and the risk of developing congenital anomalies was limited [27], which may be due to limited numbers available for study. In our study, 6,325 infants died as a result of a congenital malformation. We noted that among specific causes of infant mortality, NO_2_ and PM_10_ were associated with deaths from congenital malformations, but on closer scrutiny of specific causes of deaths, we noted differential associations. NO_2_ and PM_10_ both were associated with increased infant deaths from congenital anomalies of the nervous and gastrointestinal systems, with NO_2_ also associated with congenital anomalies of the respiratory system, whereas SO_2_ appeared to be associated with greater deaths from congenital malformations of the circulatory system. Although the mechanisms of these anomalies are likely to be difficult to identify, improvements in mortality outcomes can clearly be made by more robust environmental legislation to limit exposure, especially of pregnant women and newborn infants in infancy and childhood [28].

This study has some limitations. In particular, data on length of gestation were not available; thus, we were unable to assess the associations of the pollutants with prematurity. But we remain confident of our findings as we adjusted for birthweight, which has a robust relationship with gestation. Controlling for maternal smoking is an important consideration when conducting pollution analyses. We were unable to adjust for maternal smoking as comprehensive data were not available, nor were we able to impute these data due to the large amount of missing data, but the results are likely to remain robust as maternal smoking is closely linked with deprivation, which we adjusted for in our analyses. Our pollution exposures are based on area-based calculations that have been used previously and have been shown to be robust. Although we report the differential associations of the ambient pollutants, caution is required in interpreting the associations as the pollutants, especially NO_2_ and PM_10_, are correlated with each other. Furthermore, we associated annual averages of the pollutants with infant mortality. Since exposure in the first trimester may be most likely to be associated with congenital anomalies, and exposure after birth with infant mortality, ideally we would have liked to have associated daily or monthly exposures at critical times of development, but these are more likely to be investigated more accurately with animal models of exposures at critical periods of development [29]. Even if we had been able to study the association of mortality with pollution exposure during the different trimesters, we note that the variation in pollutant levels even within 1 week can be large, for example from 10 to 60 μg/m^3^ for NO_2_. However, although the levels of PM_10_ and NO_2_ decreased over the years of the study, there were not rapid decreases from one year to the next [30]. Pollution may be variable, being affected by other factors such as temperature and humidity. We performed simple logistic regression rather than spatiotemporal modelling; there is a small possibility that the regression may be biased due to this. Our study’s strengths are that we have studied the largest population thus far, to our knowledge, of both live births and infant deaths, which we were able to not only classify and investigate as neonatal and postneonatal deaths, but also adjust for important confounders, especially deprivation and birthweight—both great determinants of infant outcomes of mortality and morbidity.

We estimated that after adjustment for deprivation, birthweight, maternal age, infant sex, and multiple birth, when comparing quintiles 2–5 of pollution exposure to the lowest quintile, there were 261, 62, and 157 extra deaths each year due to exposure to SO_2_, NO_2_, and PM_10_. Whilst we adjusted for available confounders, others such as maternal smoking were not available. Nevertheless, it is clear that air pollutants have an association with infant mortality; thus, decreasing exposure not only for infants and children but also for pregnant mothers is a must.

In summary, we have shown that the pollutants NO_2_, PM_10_, and SO_2_ are differentially associated with neonatal and postneonatal mortality outcomes. We have also shown that these exposures are associated with different causes of infant deaths. Although progress has been made, the challenge remains to further decrease ambient pollution exposures to decrease all-cause infant mortality.

## Supporting information

S1 STROBE Checklist STROBE Statement. (DOC)

S1 Table The pollutants banded into quintiles by ranking the scores for each pollutant over each LSOA for each year (2001–2012 for NO_2_ and PM_10_ and 2002–2012 for SO_2_). (DOCX)

S2 Table Absolute numbers of deaths in each pollutant quintile. (DOCX)

S3 Table Absolute numbers for causes of infant deaths in each pollutant quintile. (DOCX)

S1 Text (DOCX)

## Abbreviations

DEFRA: Department for Environment, Food and Rural Affairs
IMD: Index of Multiple Deprivation
LSOA: Lower Layer Super Output Area
ONS: Office for National Statistics
OR: odds ratio
PM_10_: particulate matter with aerodynamic diameter ≤ 10 μm
SIDS: sudden infant death syndrome
