# Supplementary material for: Differential association of air pollution exposure with neonatal and postneonatal mortality in England and Wales: A cohort study
Source: PLoS Med. 2020 Oct 20;17(10):e1003400. doi: 10.1371/journal.pmed.1003400 (PMC7575080; doi:10.1371/journal.pmed.1003400)
Supplement: S1 STROBE Checklist — (DOC) [file pmed.1003400.s001.doc]

STROBE Statement—checklist of items that should be included in reports of observational studies

|  | Item No | Recommendation |
| --- | --- | --- |
| **Title and abstract** | 1 | 1. Indicate the study’s design with a commonly used term in the title or the abstract   ***The study design is indicate by the term “a cohort study” in the title.*** |
|  |  | 1. Provide in the abstract an informative and balanced summary of what was done and what was found   ***We believe we have provided an informative and balanced summary of what was done and what was found in the abstract.*** |
| Introduction | | |
| Background/rationale | 2 | Explain the scientific background and rationale for the investigation being reported  ***We have explained the scientific background and rationale for the investigations being reported in the introduction paragraphs 1 and 2.*** |
| Objectives | 3 | State specific objectives, including any prespecified hypotheses  ***The specific objectives and hypotheses are stated in the second paragraph of the introduction.*** |
| Methods | | |
| Study design | 4 | Present key elements of study design early in the paper  ***The key elements of study design are presented in the methods section in paragraphs 1-3.*** |
| Setting | 5 | Describe the setting, locations, and relevant dates, including periods of recruitment, exposure, follow-up, and data collection  ***The setting, locations, periods of recruitment, dates of recruitment; details on follow-up and data collection relevant to this manuscript are given in the methods section in paragraphs 1-3.*** |
| Participants | 6 | 1. *Cross-sectional study—Give the eligibility criteria, and the sources and methods of selection of participants*   ***The eligibility criteria for this study and the sources and methods of selection of participants is given in the methods section in paragraphs 1-3..*** |
|  |  |  |
| Variables | 7 | Clearly define all outcomes, exposures, predictors, potential confounders, and effect modifiers. Give diagnostic criteria, if applicable  ***All outcomes, exposures, and potential confounders are defined in the methods section in paragraphs 1-3.*** |
| Data sources/ measurement | 8* | For each variable of interest, give sources of data and details of methods of assessment (measurement). Describe comparability of assessment methods if there is more than one group  ***Sources of data and details of methods of assessment are given in the methods section in paragraphs 1-3.*** |
| Bias | 9 | Describe any efforts to address potential sources of bias  ***Any efforts to address potential sources of bias are described in the methods section in paragraphs 1-3.*** |
| Study size | 10 | Explain how the study size was arrived at  ***An explanation of the study size is given in the first paragraph of the results section.*** |
| Quantitative variables | 11 | Explain how quantitative variables were handled in the analyses. If applicable, describe which groupings were chosen and why  ***An explanation of how quantitative variables were handled in the analyses is given in the methods section in paragraphs 1-3.*** |
| Statistical methods | 12 | 1. Describe all statistical methods, including those used to control for confounding   ***All statistical methods are given in the third paragraph of the methods section. The study authorship includes an experienced medical statistician.*** |
|  |  | 1. Describe any methods used to examine subgroups and interactions   ***Any methods used to examine subgroups and interactions are given in the third paragraph of the methods section.*** |
|  |  | 1. Explain how missing data were addressed   ***There was a very very small amount of missing data (0.8%) so efforts were not made to address it.*** |
|  |  | 1. *Cross-sectional study—If applicable, describe analytical methods taking account of sampling strategy*   ***Not applicable.*** |
|  |  | 1. Describe any sensitivity analyses   ***Sensitivity analysis were not performed.*** |

Continued on next page

| Results | | |
| --- | --- | --- |
| Participants | 13* | 1. Report numbers of individuals at each stage of study—eg numbers potentially eligible, examined for eligibility, confirmed eligible, included in the study, completing follow-up, and analysed   ***The numbers of individuals at each stage of the study are reported in the first paragraph of the results section of the manuscript, and further reported in Figure 1 and the tables.*** |
|  |  | 1. Give reasons for non-participation at each stage   ***There was a minimal amount of missing data as reported in the first paragraph of the results section and in Figure 1.*** |
|  |  | 1. Consider use of a flow diagram   ***A diagram has been produced (Figure 1).*** |
| Descriptive data | 14* | 1. Give characteristics of study participants (eg demographic, clinical, social) and information on exposures and potential confounders   ***Characteristics of study participants are given in Table 1 and reported in the first paragraph of the results section.*** |
|  |  | 1. Indicate number of participants with missing data for each variable of interest   ***The number of participants with missing data for is shown in Figure 1 and in the first paragraph of the results section.*** |
|  |  |  |
| Outcome data | 15* | *Cross-sectional study—Report numbers of outcome events or summary measures*  ***Numbers of outcome events are given in the results section paragraphs 1-5 and in all the Tables in the online supp.*** |
|  |  |  |
|  |  |  |
| Main results | 16 | 1. Give unadjusted estimates and, if applicable, confounder-adjusted estimates and their precision (eg, 95% confidence interval). Make clear which confounders were adjusted for and why they were included   ***Full results are given in Tables 2-4 in the results section. It is clear which confounders are adjusted for.*** |
|  |  | 1. Report category boundaries when continuous variables were categorized   ***Category boundaries are clearly reported in the methods section paragraphs 1-3.*** |
|  |  | 1. If relevant, consider translating estimates of relative risk into absolute risk for a meaningful time period   ***Absolute risk is reported in the third and fourth paragraphs of the results section and in Online table S2.*** |
| Other analyses | 17 | Report other analyses done—eg analyses of subgroups and interactions, and sensitivity analyses  ***Sensitivity analyses were not performed.*** |
| Discussion | | |
| Key results | 18 | Summarise key results with reference to study objectives  ***Key results are summarised with reference to study objectives in the first paragraph of the discussion.*** |
| Limitations | 19 | Discuss limitations of the study, taking into account sources of potential bias or imprecision. Discuss both direction and magnitude of any potential bias  ***Limitations of the study discussed in paragraph 5 in the discussion section.*** |
| Interpretation | 20 | Give a cautious overall interpretation of results considering objectives, limitations, multiplicity of analyses, results from similar studies, and other relevant evidence  ***An overall interpretation of results is given in the discussion section paragraphs 1-6.*** |
| Generalisability | 21 | Discuss the generalisability (external validity) of the study results  ***The data is generalisabe.*** |
| Other information | | |
| Funding | 22 | Give the source of funding and the role of the funders for the present study and, if applicable, for the original study on which the present article is based  ***The sources of funding and the role of funding for the study is provided by the authors in the required forms.*** |

*Give information separately for cases and controls in case-control studies and, if applicable, for exposed and unexposed groups in cohort and cross-sectional studies.

**Note:** An Explanation and Elaboration article discusses each checklist item and gives methodological background and published examples of transparent reporting. The STROBE checklist is best used in conjunction with this article (freely available on the Web sites of PLoS Medicine at http://www.plosmedicine.org/, Annals of Internal Medicine at http://www.annals.org/, and Epidemiology at http://www.epidem.com/). Information on the STROBE Initiative is available at www.strobe-statement.org.
