# Supplementary material for: Differential association of air pollution exposure with neonatal and postneonatal mortality in England and Wales: A cohort study
Source: PLoS Med. 2020 Oct 20;17(10):e1003400. doi: 10.1371/journal.pmed.1003400 (PMC7575080; doi:10.1371/journal.pmed.1003400)
Supplement: S1 Table — (DOCX) [file pmed.1003400.s002.docx]

**ONLINE SUPPLEMENT**

**Differential association of air pollution exposure on neonatal and post-neonatal mortality in England and Wales: a cohort study**

^1^Sarah J Kotecha ,^+^ ^1^W John Watkins , ^+^ ^1^John Lowe , ^2^Jonathan Grigg , ^1^Sailesh Kotecha *

*Corresponding author

^+^Joint first authors

**S1 Table: The pollutants banded into quintiles by ranking the scores for each pollutant over each LSOA for each year (2001-2012 for NO_2_ and PM_10_ and 2002-2012 for SO_2_).**

|  | **NO_2_ (µg/m^3^)**  **Mean (range)** | **PM_10_ (µg/m^3^)**  **Mean (range)** | **SO_2_ (µg/m^3^)**  **Mean (range)** |
| --- | --- | --- | --- |
| **Number of LSOAs** | 416,671 | 417,036 | 382,283 |
| **Quintile 1** | 10.76 (2.81 – 13.91) | 13.24 (7.62 – 14.55) | 1.41 (0 – 1.79) |
| **Quintile 2** | 16.19 (13.91 – 18.18) | 15.35 (14.55 – 16.08) | 2.13 (1.79 – 2.45) |
| **Quintile 3** | 20.13 (18.18 – 22.15) | 16.78 (16.08 – 17.46) | 2.79 (2.45 – 3.15) |
| **Quintile 4** | 24.78 (22.15 – 27.83) | 18.33 (17.46 – 19.38) | 3.63 (3.15 – 4.25) |
| **Quintile 5** | 33.76 (27.83 – 66.04) | 21.86 (19.38 – 33.27) | 6.19 (4.25 – 28.81) |

LSOA – Lower Super Output Area

Numbers are mean and range
