# Supplementary material for: Differential association of air pollution exposure with neonatal and postneonatal mortality in England and Wales: A cohort study
Source: PLoS Med. 2020 Oct 20;17(10):e1003400. doi: 10.1371/journal.pmed.1003400 (PMC7575080; doi:10.1371/journal.pmed.1003400)
Supplement: S2 Table — (DOCX) [file pmed.1003400.s003.docx]

**ONLINE SUPPLEMENT**

**Differential association of air pollution exposure on neonatal and post-neonatal mortality in England and Wales: a cohort study**

^1^Sarah J Kotecha ,^+^ ^1^W John Watkins , ^+^ ^1^John Lowe , ^2^Jonathan Grigg , ^1^Sailesh Kotecha *

*Corresponding author

^+^Joint first authors

**S2 Table: Absolute numbers of deaths in each pollutant quintile.**

|  |  | **Mean (range)**  **(µg/m^3^)** | **Live births**  **N** | **Infant deaths**  **N (%)** | **Neonatal death**  **N (%)** | **Postneonatal death**  **N (%)** |
| --- | --- | --- | --- | --- | --- | --- |
| **NO_2_** | **Quintile 1** | 10.76 (2.81 – 13.91) | 1,277,198 | 4,855 (0.38) | 3,413 (0.27) | 1,442 (0.11) |
|  | **Quintile 2** | 16.19 (13.91 – 18.18) | 1,462,930 | 5,949 (0.41) | 4,142 (0.28) | 1,807 (0.12) |
|  | **Quintile 3** | 20.13 (18.18 – 22.15) | 1,567,525 | 6,694 (0.43) | 4,623 (0.29) | 2,071 (0.13) |
|  | **Quintile 4** | 24.78 (22.15 – 27.83) | 1,723,594 | 8,387 (0.49) | 5,727 (0.33) | 2,660 (0.15) |
|  | **Quintile 5** | 33.76 (27.83 – 66.04) | 1,953,119 | 10,600 (0.54) | 7,205 (0.37) | 3,395 (0.17) |
| **PM_10_** | **Quintile 1** | 13.24 (7.62 – 14.55) | 1,335,841 | 5,333 (0.40) | 3,741 (0.28) | 1,592 (0.12) |
|  | **Quintile 2** | 15.35 (14.55 – 16.08) | 1,479,377 | 6,472 (0.44) | 4,430 (0.3) | 2,042 (0.14) |
|  | **Quintile 3** | 16.78 (16.08 – 17.46) | 1,553,164 | 7,012 (0.45) | 4,832 (0.31) | 2,180 (0.14) |
|  | **Quintile 4** | 18.33 (17.46 – 19.38) | 1,690,359 | 8,141 (0.48) | 5,595 (0.33) | 2,546 (0.15) |
|  | **Quintile 5** | 21.86 (19.38 – 33.27) | 1,925,625 | 9,527 (0.49) | 6,512 (0.34) | 3,015 (0.16) |
| **SO_2_** | **Quintile 1** | 1.41 (0 – 1.79) | 1,418,838 | 5,343 (0.38) | 3,713 (0.26) | 1,630 (0.12) |
|  | **Quintile 2** | 2.13 (1.79 – 2.45) | 1,457,535 | 5,859 (0.40) | 4,076 (0.28) | 1,783 (0.12) |
|  | **Quintile 3** | 2.79 (2.45 – 3.15) | 1,507,333 | 6,837 (0.45) | 4,681 (0.31) | 2,156 (0.14) |
|  | **Quintile 4** | 3.63 (3.15 – 4.25) | 1,523,899 | 7,560 (0.50) | 5,170 (0.34) | 2,390 (0.16) |
|  | **Quintile 5** | 6.19 (4.25 – 28.81) | 1,484,008 | 7,805 (0.53) | 5,371 (0.36) | 2,434 (0.16) |

Numbers are mean and range
