# Supplementary material for: Differential association of air pollution exposure with neonatal and postneonatal mortality in England and Wales: A cohort study
Source: PLoS Med. 2020 Oct 20;17(10):e1003400. doi: 10.1371/journal.pmed.1003400 (PMC7575080; doi:10.1371/journal.pmed.1003400)
Supplement: S3 Table — (DOCX) [file pmed.1003400.s004.docx]

**ONLINE SUPPLEMENT**

**Differential association of air pollution exposure on neonatal and post-neonatal mortality in England and Wales: a cohort study**

^1^Sarah J Kotecha ,^+^ ^1^W John Watkins , ^+^ ^1^John Lowe , ^2^Jonathan Grigg , ^1^Sailesh Kotecha *

*Corresponding author

^+^Joint first authors

**S3 Table: Absolute numbers for causes of infant deaths in each pollutant quintile.**

|  |  | **Infant births** | **Perinatal N (%)** | **Congénital malformations N (%)** | **Respiratory N (%)** | **Neoplasm N (%)** | **Endocrine N (%)** | **Blood N (%)** | **Other N (%)** |
| --- | --- | --- | --- | --- | --- | --- | --- | --- | --- |
| **NO2_Band** | 1 | 1,277,198 | 2,915 (0.228) | 804 (0.063) | 95 (0.007) | 35 (0.003) | 43 (0.003) | 8 (0.001) | 955 (0.075) |
|  | 2 | 1,462,930 | 3,563 (0.244) | 967 (0.066) | 122 (0.008) | 40 (0.003) | 66 (0.005) | 14 (0.001) | 1,177 (0.08) |
|  | 3 | 1,567,525 | 3,938 (0.251) | 1,118 (0.071) | 158 (0.01) | 41 (0.003) | 101 (0.006) | 15 (0.001) | 1,323 (0.084) |
|  | 4 | 1,723,594 | 4,852 (0.282) | 1,503 (0.087) | 200 (0.012) | 45 (0.003) | 144 (0.008) | 29 (0.002) | 1,614 (0.094) |
|  | 5 | 1,953,119 | 6,228 (0.319) | 1,933 (0.099) | 217 (0.011) | 51 (0.003) | 193 (0.01) | 45 (0.002) | 1,933 (0.099) |
|  | Total | 7,984,366 | 21,496 | 6,325 | 792 | 212 | 547 | 111 | 7002 |
| **PM10_Band** | 1 | 1,335,841 | 3,158 (0.236) | 903 (0.068) | 91 (0.007) | 31 (0.002) | 63 (0.005) | 12 (0.001) | 1,075 (0.08) |
|  | 2 | 1,479,377 | 3,738 (0.253) | 1,147 (0.078) | 157 (0.011) | 44 (0.003) | 108 (0.007) | 14 (0.001) | 1,264 (0.085) |
|  | 3 | 1,553,164 | 4,089 (0.263) | 1,211 (0.078) | 154 (0.01) | 38 (0.002) | 95 (0.006) | 17 (0.001) | 1,408 (0.091) |
|  | 4 | 1,690,359 | 4,869 (0.288) | 1,330 (0.079) | 187 (0.011) | 46 (0.003) | 127 (0.008) | 31 (0.002) | 1,551 (0.092) |
|  | 5 | 1,925,625 | 5,642 (0.293) | 1,734 (0.09) | 203 (0.011) | 53 (0.003) | 154 (0.008) | 37 (0.002) | 1,704 (0.088) |
|  | Total | 7,984,366 | 21,496 | 6,325 | 792 | 212 | 547 | 111 | 7,002 |
| **SO2_Band** | 1 | 1,418,838 | 3,183 (0.224) | 948 (0.067) | 109 (0.008) | 41 (0.003) | 63 (0.004) | 23 (0.002) | 976 (0.069) |
|  | 2 | 1,457,535 | 3,534 (0.242) | 974 (0.067) | 114 (0.008) | 43 (0.003) | 75 (0.005) | 11 (0.001) | 1,108 (0.076) |
|  | 3 | 1,507,333 | 4,023 (0.267) | 1,193 (0.079) | 142 (0.009) | 41 (0.003) | 109 (0.007) | 17 (0.001) | 1,312 (0.087) |
|  | 4 | 1,523,899 | 4,381 (0.287) | 1,409 (0.092) | 180 (0.012) | 37 (0.002) | 128 (0.008) | 30 (0.002) | 1,395 (0.092) |
|  | 5 | 1,484,008 | 4,574 (0.308) | 1,295 (0.087) | 172 (0.012) | 37 (0.002) | 129 (0.009) | 23 (0.002) | 1,575 (0.106) |
|  | Total | 7,391,613 | 19,695 | 5,819 | 717 | 199 | 504 | 104 | 6,366 |

**Numbers are total number and percentages.**
