# Supplementary material for: Differential association of air pollution exposure with neonatal and postneonatal mortality in England and Wales: A cohort study
Source: PLoS Med. 2020 Oct 20;17(10):e1003400. doi: 10.1371/journal.pmed.1003400 (PMC7575080; doi:10.1371/journal.pmed.1003400)
Supplement: S1 Text — (DOCX) [file pmed.1003400.s005.docx]

S1 Text

1. **Title of the Research Project:***

| The effect of pollution on child mortality, (stillbirths and deaths under the age of 19 years). |
| --- |

1. **Abstract of the Research Project:***

*Please include a short description of the project and its benefits,*

| Our initial project which has successfully completed investigated the rates of death in childhood and adolescence after low birthweight (published in PLoS Medicine 2016). We have successfully secured approval of extending this last project to investigate the role of deprivation on death in childhood after low birthweight, etc. We have also submitted another application which will study the effect of pollution on stillbirths, IUGR, rates of prematurity, and deaths under the age of 19 years. We would like to amend this last application to enable us to carry out part of the analysis whilst the full application is under consideration by NHS Digital. We seek to investigate the association between pollution and stillbirths, and risk of death in children under the age of 19 years including neonatal and infant deaths. We additionally want to be able to account for deprivation and other factors in this analysis and look at causes of death so that independent effects of pollution in death rates can be attributed. We will link the data to pollution data from DEFRA. The benefits of this project are the research will inform clinicians and policy makers of the impact of pollution on rates of stillbirths, and risk of death in children under the age of 19 years.  Since there is considerable overlap between this amendment and the already approved project we would like to request the data only once to avoid using your resources on multiple occasions. |
| --- |

1. **Details of Research Project:**
   1. *Please provide a detailed description of the purpose for which the data are requested, describing the aims of the study/research. Where research is part of a larger programme, please include details below;*

| We have a strong background in the investigation of on infant mortality (death before 1 year) and as well as journal publications we produce the All Wales Perinatal Survey (AWPS) report every year that gives the last year’s rates and figures and compares those to previous years. We are particularly interested in the effects of pollution on these outcomes.  We seek to investigate the association between pollution and rates of stillbirths, and risk of death in children under the age of 19 years. We additionally want to be able to account for deprivation and other factors in this analysis and look at causes of death. We will link the data to pollution data from DEFRA. The benefits of this project are the research will inform clinicians and policy makers of the impact of pollution on rates of stillbirths, and risk of death in children under the age of 19 years. |
| --- |

1. **Data Required:**

| We would like to link the ONS LSOA data that we have requested to pollutant data at LSOA level for data obtained from DEFRA to study the effect of pollutants exposure on important perinatal outcomes including stillbirths, neonatal and child/adolescent deaths. |
| --- |
